# Longitudinal Radiological Remodeling after Irreversible Electroporation Versus Radiofrequency Ablation: A Prospective Comparative Study (The LIRA Study)

**DOI:** 10.7150/ijms.133300

**Published:** 2026-03-17

**Authors:** Yiting Liu, Qin Liu, Haiyang Yu, Wei Huang, Ziyin Wang, Qingbing Wang, Jingjing Liu, Xiaoyan Fei, Junwei Gu, Zhongmin Wang, Xiaoyi Ding, Zhiyuan Wu

**Affiliations:** 1 Department of Interventional Radiology, Ruijin Hospital, Shanghai Jiao Tong University School of Medicine, No. 197, Ruijin Er Road, Shanghai, China.; 2 Faculty of Medical Imaging Technology, College of Health Science and Technology, Shanghai Jiao Tong University School of Medicine, Shanghai, China.; 3 Liver Center, Ruijin Hospital, Shanghai Jiao Tong University School of Medicine, Shanghai 200025, China.

**Keywords:** irreversible electroporation, radiofrequency ablation, liver tumors, ablation zone, radiological evolution, local tumor control

## Abstract

**Background:**

To evaluate the short- to intermediate-term radiological evolution of ablation zones using contrast-enhanced MRI and to assess clinically relevant outcomes following irreversible electroporation (IRE) and radiofrequency ablation (RFA) in patients with liver tumors.

**Methods:**

Thirty-five ablation zones from 24 patients were prospectively evaluated in the LIRA Study. Serial contrast-enhanced MRI was performed at predefined time points (post-ablation day 7 as baseline, 1 month, and 3 months), and ablation-zone geometry was quantitatively analyzed and compared between groups. In addition to longitudinal radiological evolution of the ablation zone, local recurrence-free survival (LRFS) was evaluated as the primary clinical endpoint; disease control and treatment-related safety were also assessed. Peripheral immune profiling was assessed in an exploratory manner.

**Results:**

Longitudinal imaging demonstrated distinct ablation-zone remodeling patterns between the two modalities. Ablation-zone volume and surface area decreased significantly at both 1 and 3 months in both groups, with smaller normalized values in the IRE group. In contrast, a significantly greater change in the surface area-to-volume ratio was observed only at 3 months with IRE versus RFA (IRE: 0.27 [0.16-0.36] vs. RFA: 0.04 [0.02-0.08]; *p* < 0.001). Treatment efficacy was comparable between groups, whereas procedure-related complications occurred more frequently in the IRE group (45% vs. 0%; *p* = 0.011). Exploratory peripheral immune profiling revealed time-dependent immune fluctuations following IRE.

**Conclusions:**

IRE is associated with more pronounced longitudinal radiological evolution of the ablation zone compared with RFA, with comparable clinical outcomes and time-dependent immune fluctuations observed after ablation in liver tumors.

## Introduction

Ablation techniques are widely used in the treatment of liver tumors, particularly in patients with early-stage disease or those who are not candidates for surgical resection 1. Radiofrequency ablation (RFA) induces tissue destruction through thermal coagulative necrosis and remains the most commonly applied ablation modality, with broad support from current clinical guidelines 2. In contrast, irreversible electroporation (IRE) is a non-thermal ablation technique that delivers high-voltage electric pulses to permeabilize cell membranes and induce cell death, while largely preserving the extracellular matrix and adjacent vascular and biliary structures 3, 4.

Owing to these fundamental differences in the mechanisms of tissue injury, thermal and non-thermal ablation are expected to elicit distinct inflammatory responses and subsequent repair and remodeling processes. After thermal ablation, tissue injury is primarily characterized by coagulative necrosis, followed by a prolonged repair phase marked by progressive fibrotic remodeling and the development of a peripheral reaction zone in the adjacent liver parenchyma 5. In contrast, ablation zones after IRE undergo a distinct healing process, with preservation of the extracellular matrix and vascular scaffolding. On imaging, post-IRE ablation zones are typically characterized by a well-demarcated hypoattenuating central area that gradually contracts and may become less conspicuous over time 6. These differences in tissue injury and repair suggest that the temporal evolution of ablation zones, as well as their clinical and biological implications, may differ substantially between thermal and non-thermal ablation. Although IRE has gained increasing attention as a safe and feasible option for treating liver tumors, particularly those located near major vascular structures, direct longitudinal comparisons with RFA remain limited 7. In particular, systematic evaluations integrating post-ablation tissue remodeling and longitudinal imaging evolution with treatment efficacy, safety, and immune-related changes are lacking.

Therefore, in this prospective exploratory study, we primarily focused on a longitudinal imaging-based comparison of ablation zone evolution following RFA and IRE. Using serial contrast-enhanced MRI and three-dimensional manual segmentation, we quantitatively assessed changes in ablation-zone volume, surface area, and geometry across predefined follow-up time points. In addition to this imaging-based analysis, clinically relevant outcomes, including treatment efficacy and safety, were systematically evaluated between ablation approaches. Local recurrence-free survival (LRFS) was used as a key indicator of local tumor control, along with assessment of treatment-related complications, disease control, and survival outcomes. Longitudinal peripheral immune profiles were additionally explored to provide complementary biological information alongside the imaging analysis.

## Methods

### Study protocol

**Figure 1:**
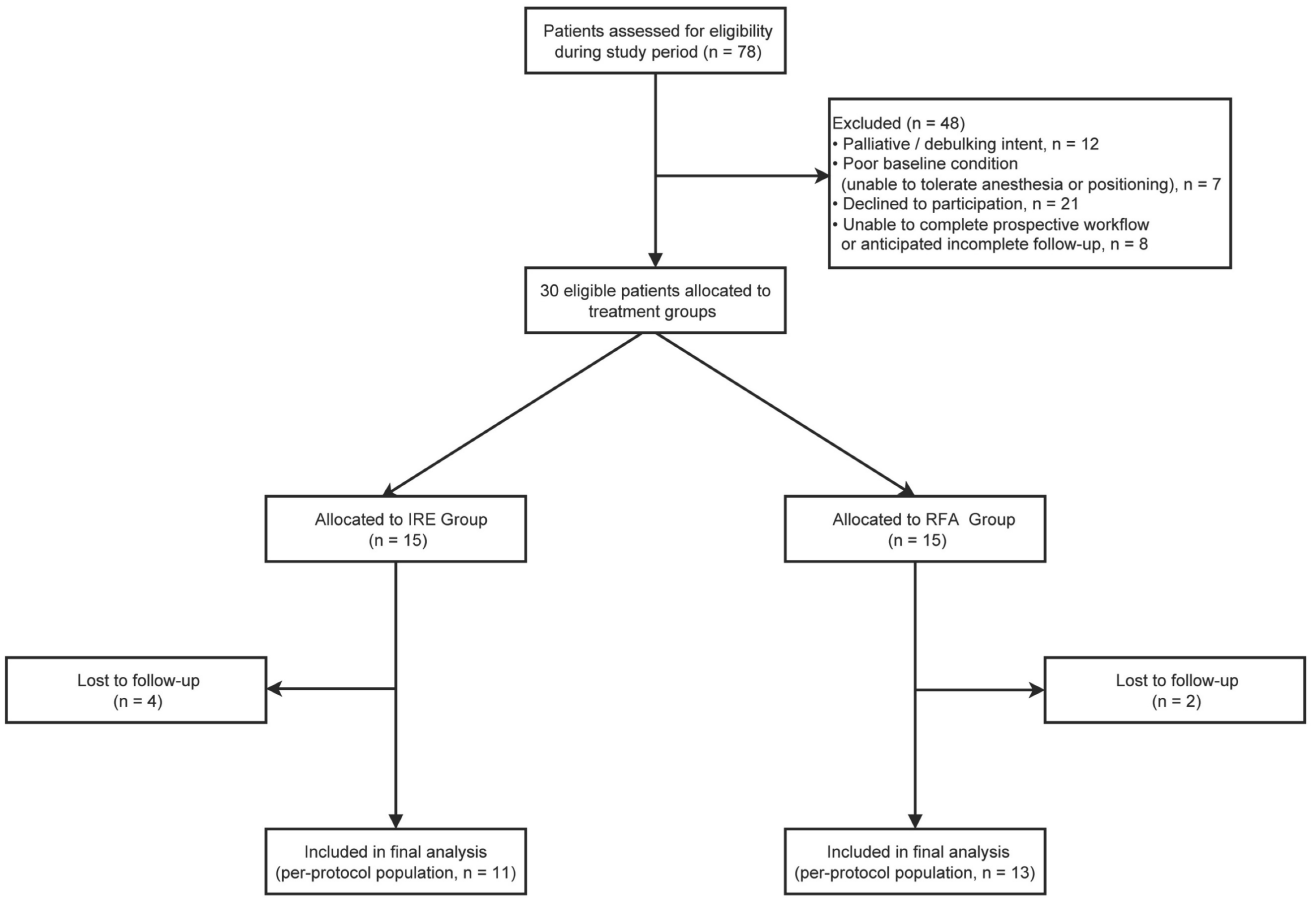
Flow diagram of patient enrollment, group allocation, and follow-up in this prospective study.

This prospective randomized study (The LIRA Study) was approved by the Ethics Committee of Ruijin Hospital, Shanghai Jiao Tong University School of Medicine (Approval No. 86/2018). Written informed consent was obtained from all participants prior to enrollment. Between May 2019 and December 2022, patients with primary or secondary liver tumors who were unsuitable for or declined surgical resection were prospectively enrolled. Inclusion criteria were age 18-80 years; up to five hepatic lesions, with a maximum diameter of 30 mm for any single lesion; and an Eastern Cooperative Oncology Group performance status of 0-2. Exclusion criteria included palliative or debulking-intent treatment; poor general condition, such as severe cardiopulmonary insufficiency precluding tolerance of anesthesia or procedural positioning; insufficient preoperative baseline data or inability to complete the prospective workflow; and refusal to participate in the study. During the study period, 78 patients were assessed for eligibility. After exclusion of patients who did not meet the predefined criteria or declined participation, 30 eligible patients were randomized in a 1:1 ratio to the IRE group (n = 15) or the RFA group (n = 15) using sealed envelopes. During follow-up, 6 patients were lost to follow-up, and the final per-protocol analysis included 24 patients (IRE, n = 11; RFA, n = 13) (Figure 1).

### Patient and Treatment Characteristics

Baseline demographic, clinical, and tumor characteristics of the final per-protocol population are summarized in Table 1. A total of 35 target lesions in 24 patients were included in the analysis, with 11 patients (n = 12 lesions) treated with IRE and 13 patients (n = 23 lesions) treated with RFA. The cohort comprised 16 men and 8 women, with a mean age of 57.5 ± 8.3 years. The most common tumor types were hepatocellular carcinoma (HCC; n = 12) and metastatic colorectal cancer (n = 7). Most patients presented with solitary lesions (63%, 15/24). The mean tumor diameter was 18.4 ± 6.6 mm in the IRE group and 19.4 ± 10.1 mm in the RFA group. Four patients in each group had previously undergone surgery, whereas chemotherapy was administered to two patients in the IRE group and three patients in the RFA group. High-risk locations were defined as tumors located within 5 mm of major vessels, bile ducts, or hollow viscera.

### Ablation procedure

The choice of ablation modality was determined by randomization, and all IRE and RFA procedures were performed by the same two experienced interventional radiologists according to standardized institutional protocols. Patients underwent general anesthesia with neuromuscular blockade.

RFA was performed using a Covidien 11c RF generator (Covidien, USA) in combination with StarBurst XL expandable electrodes (AngioDynamics, Latham, NY, USA). The electrodes were percutaneously inserted into the target lesion under computed tomography (CT) guidance according to the preprocedural treatment plan. Ablation power and duration were determined by the operators to ensure adequate coverage of the lesion. To minimize the risk of tumor seeding, track ablation was routinely performed during needle withdrawal. IRE was performed using the NanoKnife system (Intelligent HealthMedical Co., Ltd, Tianjin, China and AngioDynamics, Latham, NY, USA) under CT guidance with electrocardiogram (ECG) synchronization to minimize the risk of procedure-related arrhythmias during pulse delivery. Nineteen-gauge monopolar electrodes were percutaneously inserted in a parallel configuration to encompass the target lesion, with an inter-electrode spacing of 1.2-2.2 cm and an exposed electrode tip length of 1-2 cm.

After electrode placement, 10-20 test pulses were routinely delivered to verify adequate current transmission prior to full pulse delivery. A current range of approximately 25-45 A was considered acceptable. If the measured current was outside this range or impedance feedback was suboptimal, pulse parameters were adjusted as needed, and electrode repositioning was performed if required. Once adequate electrical conditions were confirmed, ablation was carried out using standard parameters, including an electric field strength of approximately 1500 V/cm, a pulse duration of 70-90 μs, and 90 pulses per ablation cycle. The number of electrodes and ablation parameters were adjusted according to tumor size and location to ensure complete coverage of the lesion with a safety margin of at least 5 mm.

### Assessment of follow-up

To assess ablation-related bleeding and other procedure-related complications, contrast-enhanced MRI was performed 1 day after ablation in both the RFA and IRE groups. Follow-up contrast-enhanced MRI was subsequently performed at 7 days and 1 month after the procedure to evaluate technical success. Thereafter, imaging follow-up was conducted at 3-month intervals and continued at 6-month intervals after 2 years.

Technical success was defined as complete coverage of the target lesion by the ablation zone without residual enhancement on contrast-enhanced MRI obtained 7 days after ablation. All post-ablation images were evaluated for local tumor control. Local tumor progression (LTP) was defined as the presence of contrast-enhancing tumor foci within 1 cm of the ablation zone. LRFS was defined as the time from ablation to local tumor progression or death from any cause and was designated as the primary endpoint. Secondary outcomes included progression-free survival (PFS), objective response rate (ORR), overall survival (OS), and safety. PFS was assessed according to modified Response Evaluation Criteria in Solid Tumors (mRECIST) and measured from ablation to tumor progression or death. ORR was defined as the proportion of patients achieving complete or partial response during follow-up. Overall survival (OS) was calculated from ablation to death from any cause 8. Patients without events were censored at the date of last follow-up. To assess procedural safety, ablation-related complications were recorded and graded according to the Society of Interventional Radiology (SIR) classification system 9. Ablation zones were manually delineated by a radiologist using 3D Slicer software based on the portal venous phase images. All radiological assessments were performed by the same radiologist with over 15 years of experience in abdominal imaging.

Peripheral blood samples were collected at baseline (before ablation) and at 1, 3, 7, 14, and 28 days after ablation for routine hematological testing and peripheral immune cell analysis by flow cytometry.

### MRI acquisition protocol

All MRI examinations were performed using one of three 3.0-T whole-body MR systems: SIGNA Architect AIR (GE Medical Systems, USA), uMR 790 (United Imaging Healthcare, China), or Ingenia (Philips Medical Systems, the Netherlands), each equipped with dedicated phased-array body coils. A standard dose of gadopentetate dimeglumine (0.1 mmol/kg body weight; Magnevist®, Bayer AG, Germany) was administered intravenously. Contrast-enhanced MRI included pre-contrast T1-weighted imaging and three-phase dynamic acquisitions (arterial, portal venous, and delayed phases). Dynamic contrast-enhanced imaging was performed using breath-hold three-dimensional T1-weighted gradient-echo sequences, with vendor-specific implementations adapted to each MR system: the LAVA sequence on the GE scanner, the t1_quick3d sequence on the United Imaging scanner, and the mDIXON-W sequence on the Philips scanner.

### Statistical analysis

Statistical analyses and data visualization were performed using R software (version 4.3.2). LRFS, PFS, OS were estimated by the Kaplan-Meier method and compared between groups using the log-rank test. Categorical variables were compared using Fisher's exact test. Continuous variables are presented as mean ± standard deviation for approximately normally distributed data and as median with interquartile range (IQR) for non-normally distributed data. Comparisons of continuous variables between groups were performed using the two-sided Student's t-test when normality and homogeneity of variance were satisfied, the Welch's t-test when variance homogeneity was violated, or the Wilcoxon rank-sum test for non-normally distributed data. Volumetric and surface area measurements of the ablation zone at 1month and 3 months were normalized to the 7-days post-ablation baseline. All statistical tests were two-sided, and a *p*-value < 0.05 was considered statistically significant. Statistical significance was denoted as *p* < 0.05 (*), *p* < 0.01 (**), and *p* < 0.001 (***).

## Results

### Comparison of Post-Ablation Zone Evolution Between IRE and RFA

**Figure 2:**
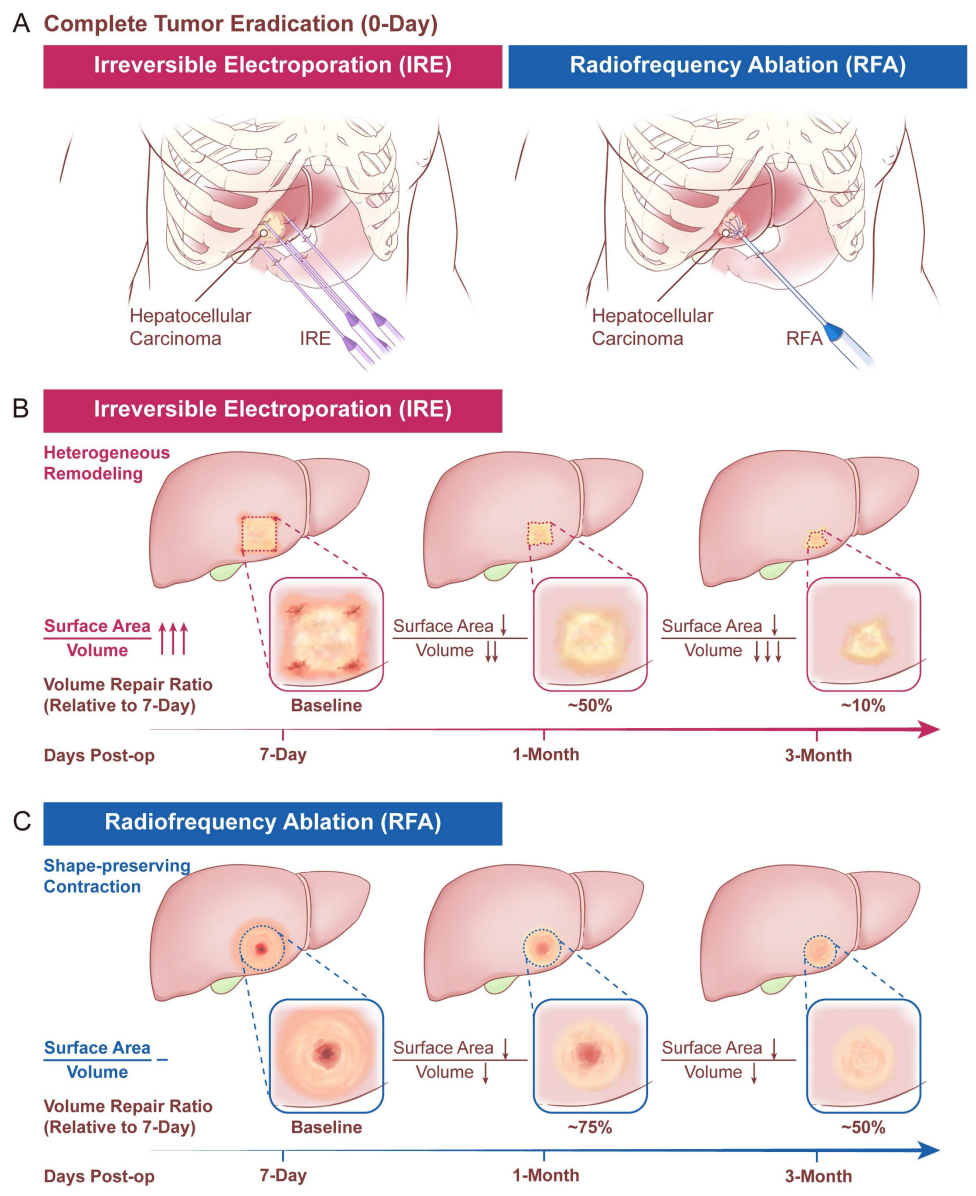
** Schematic illustration of the longitudinal radiological assessment of ablation zone evolution after IRE and RFA. (A)** Conceptual depiction of tumor ablation using IRE and RFA; **(B, C)** Representative schematic illustrations of longitudinal morphologic evolution of the ablation zone following IRE **(B)** and RFA **(C)** during follow-up.

**Figure 3:**
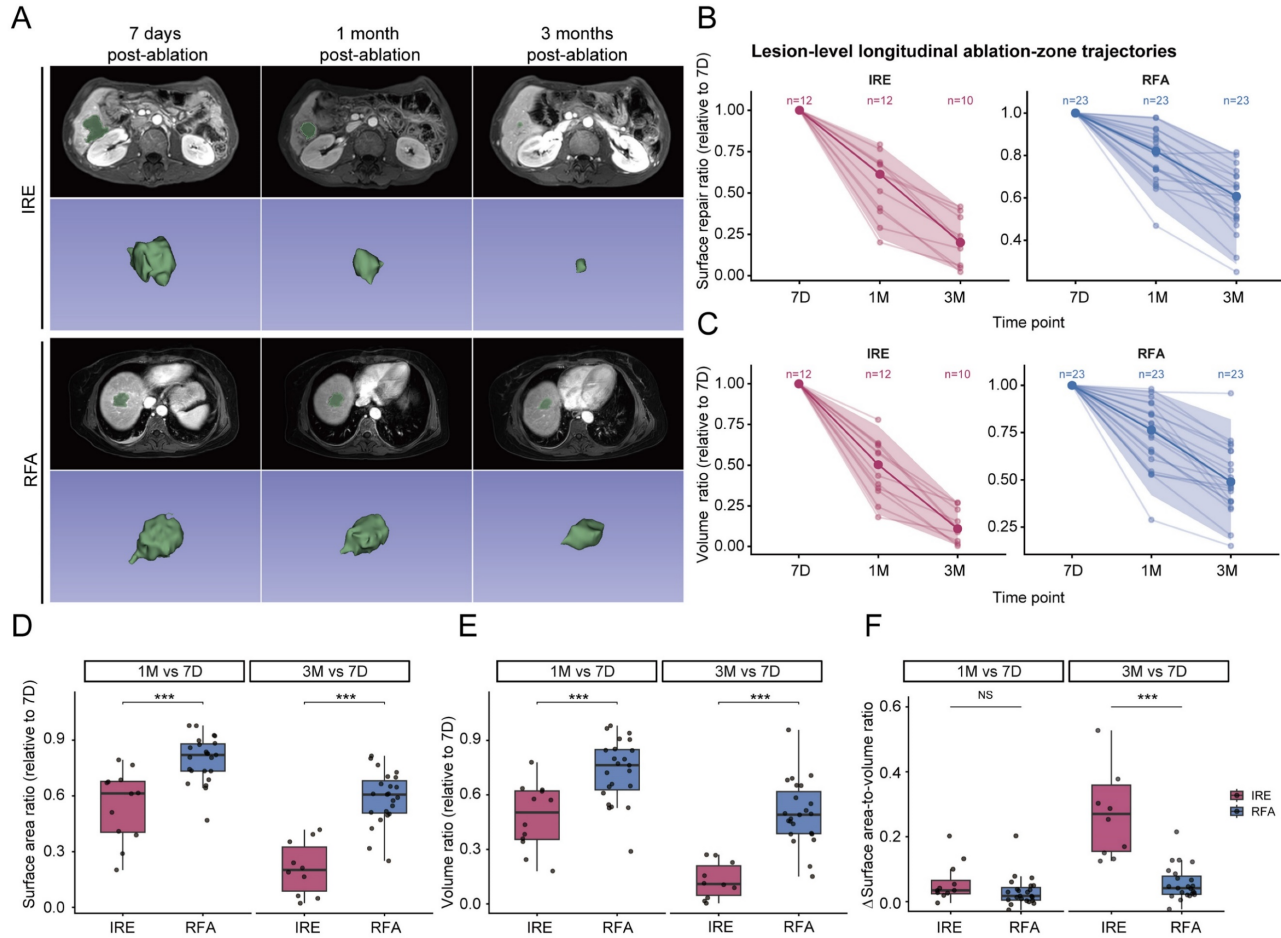
** Quantitative analysis of longitudinal changes in ablation-zone morphology following IRE and RFA. (A)** Representative axial and three-dimensional (3D) reconstructions of portal venous-phase MRI images obtained at predefined follow-up time points after IRE and RFA; **(B, C)** Lesion-level longitudinal trajectories of normalized ablation-zone surface area **(B)** and volume ratio **(C)**, relative to the 7-day post-procedure baseline; **(D, E)** Comparison of normalized ablation-zone surface area ratio **(D)** and volume ratio **(E)** between the IRE and RFA groups at 1 month and 3 months after ablation; **(F)** Longitudinal changes in the surface area-to-volume ratio (the difference (Δ) from the 7-day baseline) in the two treatment modalities. Statistical comparisons between groups were performed using the Wilcoxon rank-sum test (*p < 0.05, **p < 0.01, ***p < 0.001).

The procedural schematics of the two ablation modalities (IRE and RFA) are shown in Figure 2A, and the conceptual morphologic evolution of the ablation zone at predefined follow-up time points is shown in Figure 2B and 2C. Quantitative measurements of ablation-zone surface area and volume at each follow-up time point are summarized in Table 2. At 7 days after ablation, no significant difference was observed in ablation-zone surface area between the two groups, whereas ablation-zone volume was smaller in the IRE group. During longitudinal follow-up, both surface area and volume progressively decreased in both groups (Figure 3A).

For comparative analysis of ablation-zone regression trends, measurements were normalized to the 7-day post-ablation baseline. After normalization, residual ablation-zone surface area and volume fractions at 1 and 3 months were significantly lower in the IRE group than in the RFA group. Specifically, in the IRE group, relative surface area decreased from 0.61 to 0.20 and relative volume from 0.5 to 0.11, whereas corresponding values in the RFA group decreased from 0.82 to 0.61 for surface area and from 0.76 to 0.49 for volume (Figure 3B-3E and Table 2). To further characterize morphological changes of the ablation zone, changes in the surface area-to-volume ratio relative to the 7-day baseline were compared between modalities. No significant difference was observed at 1 month after ablation. In contrast, a significant difference emerged at 3 months, with a higher surface area-to-volume ratio in the IRE group than in the RFA group (IRE vs. RFA: 0.27 [0.16-0.36] vs. 0.04 [0.02-0.08], median [Q1-Q3]; *p* < 0.001) (Figure 3F). These results indicate that ablation-zone evolution differs between the two modalities not only in the magnitude of size reduction but also in geometric characteristic.

### Assessment of treatment outcome

**Figure 4:**
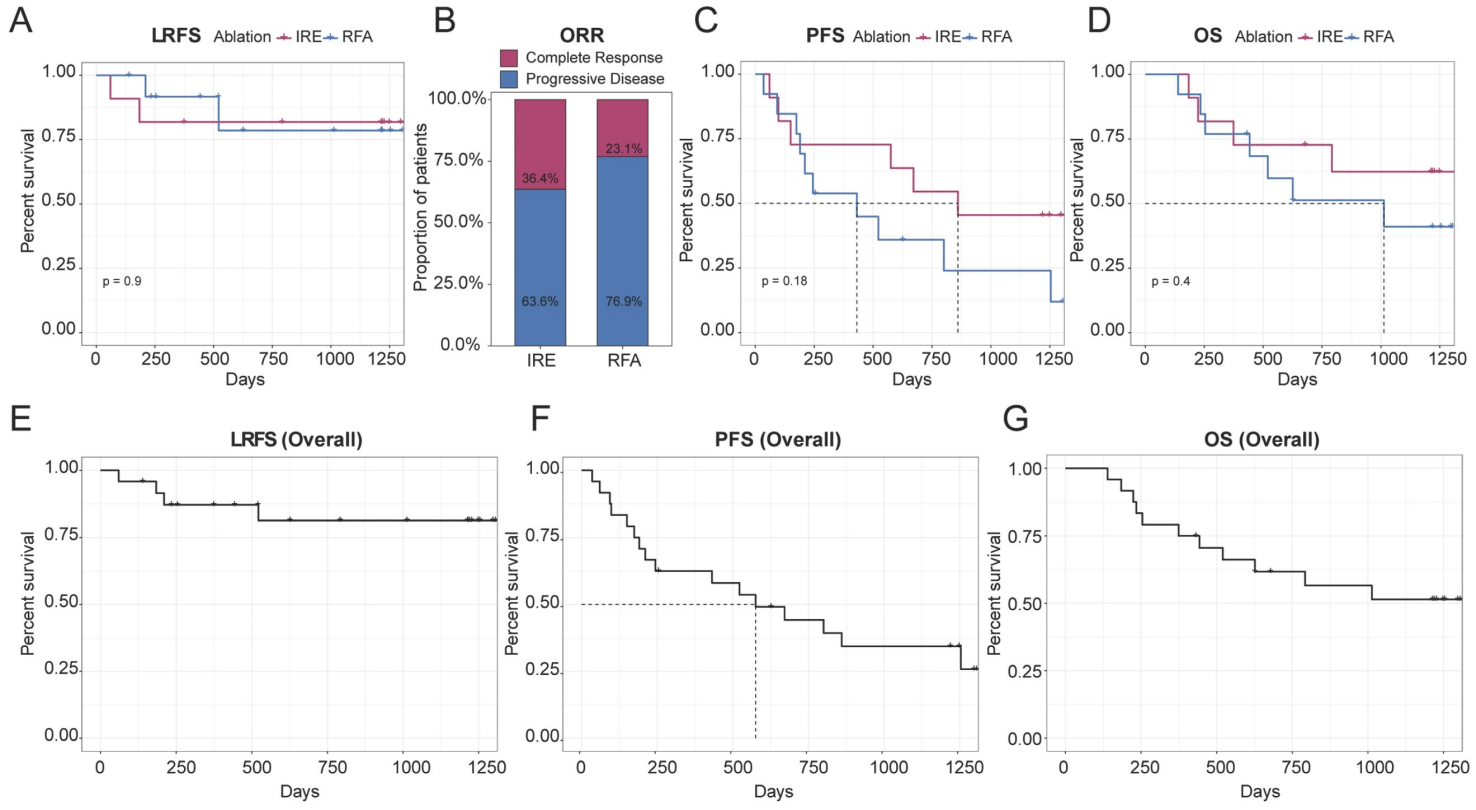
** Clinical outcomes following IRE and RFA. (A)** Kaplan-Meier analysis of LRFS comparing the IRE and RFA groups; **(B)** ORR at the last available follow-up in the IRE and RFA groups, assessed according to mRECIST criteria; **(C, D)** Kaplan-Meier analyses of PFS **(C)** and OS **(D)** comparing the two treatment groups; **(E-G)** Kaplan-Meier curves showing overall LRFS **(E)**, PFS **(F)**, and OS **(G)** in the entire study cohort.

**Figure 5:**
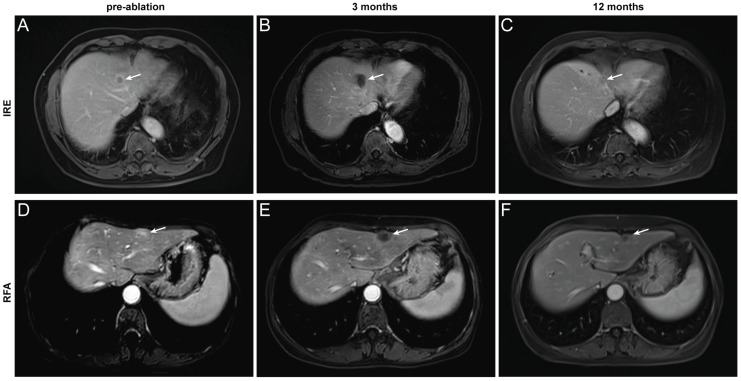
** Representative contrast-enhanced MRI images illustrating complete tumor ablation after IRE and RFA. (A-C)** A 67-year-old man with hepatocellular carcinoma treated with IRE. Images obtained before ablation (A, delayed phase) and at 3 months **(B)** and 12 months **(C)** after ablation (portal venous phase); **(D-F)** A 64-year-old woman with colorectal liver metastasis treated with RFA. Images obtained before ablation (D, delayed phase) and at 3 months **(E)** and 12 months **(F)** after ablation (portal venous phase). The target lesion and ablation zone are indicated by arrows.

Based on the distinct ablation-zone remodeling patterns observed between IRE and RFA, clinical outcomes were further evaluated. LTP occurred in four patients after ablation, including three patients with secondary liver tumors and one patient with primary liver cancer (Table S1). The patient-level LTP rates were 18% in the IRE group and 15% in the RFA group (Table 3). For the primary endpoint, the median follow-up duration for LRFS was 40.5 months (range, 2-44.2 months). Kaplan-Meier analysis demonstrated no significant difference in LRFS between the IRE and RFA groups (Figure 4A). The LRFS rates at 6 months, 1, and 3 years were 90.9%, 81.8%, and 81.8% in the IRE group, respectively, and 100%, 91.7%, and 78.6% at the corresponding time points in the RFA group. Median LRFS was not reached in either group during the follow-up period. The overall ORR was 36.4% in the IRE group and 23.1% in the RFA group (Figure 4B). No significant differences were observed in PFS or OS between the two groups (Figure 4C, D). Representative cases are shown in Figure 5. Pre-ablation delayed-phase images depict the target lesions treated with IRE and RFA (Figure 5A and D). During follow-up, venous-phase images demonstrate well-demarcated hypoenhancing ablation zones at 3 months (Figure 5B and E) and 12 months (Figure 5C and F) after ablation, indicating complete lesion eradication.

**Figure 6:**
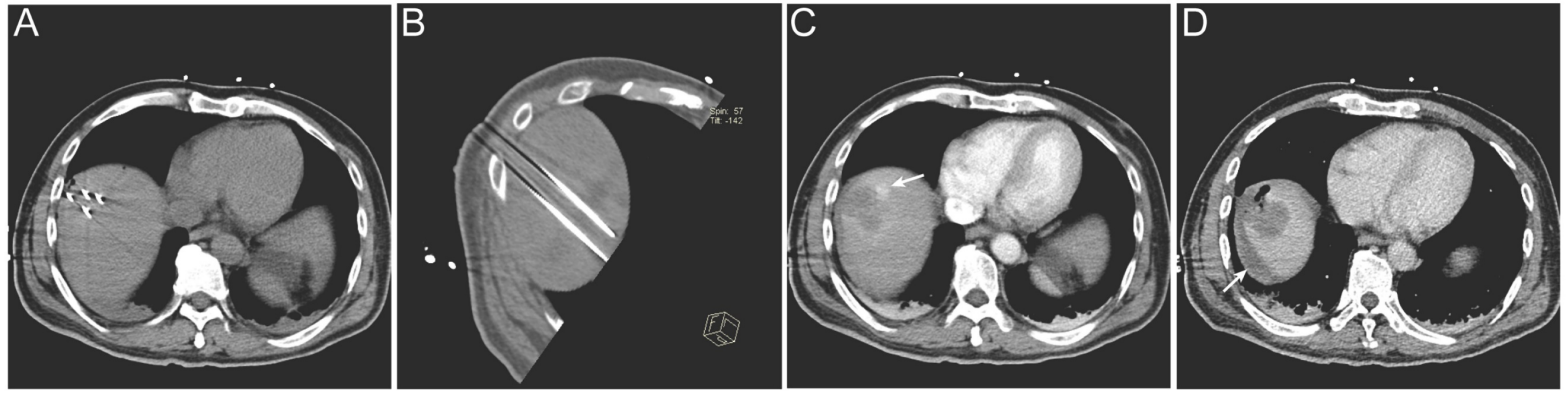
** Representative imaging of procedure-related complication following IRE treatment. (A, B)** Intra-procedural electrode placement shown on an axial CT image **(A)** and an oblique multiplanar reconstructed (MPR) CT image **(B)**; **(C)** Immediate post-ablation arterial-phase contrast-enhanced CT demonstrating focal contrast extravasation at the ablation margin (arrow); **(D)** Post-ablation delayed-phase contrast-enhanced CT demonstrating perilesional fluid attenuation (arrow).

Lesions located in high-risk anatomical regions were comparably distributed between the two groups, with 7 lesions in the IRE group and 6 lesions in the RFA group; however, lesions in the IRE group were more frequently adjacent to major vessels or the hepatic hilum (Table 4). Regarding treatment safety, no deaths within 30 days after treatment were recorded and no severe complications were observed in the RFA group. In the IRE group, one patient developed ablation-related bile duct dilatation and four patients experienced hemorrhage. All adverse events were successfully managed with routine symptomatic treatment (Table 3). A representative case is shown in Figure 6. Intra-procedural images demonstrate electrode placement (Figure 6A, B). Post-ablation arterial-phase imaging demonstrates focal contrast extravasation at the ablation margin, and perilesional fluid attenuation is observed on delayed-phase imaging (Figure 6C, D).

### Longitudinal changes in peripheral immune profiles following ablation

Peripheral immune-related blood parameters were summarized to reflect systemic immune changes after ablation (Table S2). Both ablation modalities exhibited transient post-procedural increases in leukocyte and neutrophil counts, as well as the neutrophil-to-lymphocyte ratio (NLR), peaking on day 1 and gradually returning toward baseline thereafter. However, peripheral lymphocyte counts underwent slight fluctuation. Overall, the temporal patterns of these immune-related blood parameters appeared comparable between the IRE and RFA groups.

**Figure 7:**
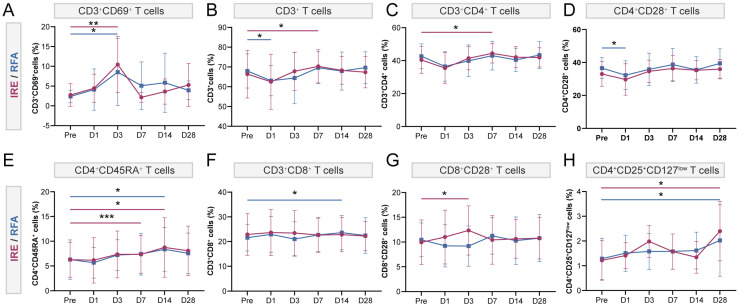
** Longitudinal changes in peripheral T-cell subsets following IRE (red) or RFA (blue). Peripheral** blood T-cell subsets were analyzed by flow cytometry at baseline (Pre) and at 1, 3, 7, 14, and 28 days after ablation. (A-H) Proportions of **(A)** CD3^+^CD69^+^ activated T cells; **(B)** CD3^+^ T cells; **(C)** CD3^+^CD4^+^ T cells; **(D)** CD4^+^CD28^+^ T cells; **(E)** CD4^+^CD45RA^+^ T cells; **(F)** CD3^+^CD8^+^ T cells; **(G)** CD8^+^CD28^+^ T cells; and **(H)** CD4^+^CD25^+^CD127^low^ regulatory T cells (Tregs). Data are presented as mean ± SD. Within-group comparisons versus baseline were performed using paired t-tests. *P < 0.05, **P < 0.01, ***P < 0.001 vs baseline.

To further characterize immune alterations beyond routine blood parameters, longitudinal immune profiling of peripheral blood was performed using flow cytometry. Compared with pre-ablation baseline levels, both ablation modalities induced early immune activation, as reflected by increased proportions of activated T cells (CD3^+^CD69^+^) on day 3. Specifically, activated T cells were significantly elevated on day 3 in both the IRE group (10.45 ± 7.11% vs. 2.74 ± 2.88% at baseline, *p* < 0.01) and the RFA group (8.53 ± 8.46% vs. 2.38 ± 1.49%, *p* < 0.05) (Figure 7A). In the RFA group, immune cell proportions showed fluctuating patterns: CD3⁺ T cells, CD3^+^CD4^+^ T cells, and CD4^+^CD28^+^ T cells initially decreased after ablation and subsequently returned toward baseline levels (Figure 7B, C, D). Naïve CD4^+^ T cells (CD4^+^CD45RA^+^) and CD3^+^CD8^+^ T cells also exhibited dynamic changes, reaching their highest levels around day 14 after ablation (Figure 7E, F). Despite only minor fluctuations in CD4^+^CD28^+^ and CD3^+^CD8^+^ T cells (Figure 7D, F), the IRE group displayed a more pronounced immune-activated status during days 3-7, characterized by increased proportions of CD3^+^ T cells, CD3^+^CD4^+^ T cells, naïve CD4^+^ T cells (CD4^+^CD45RA^+^) and activated CD8^+^ T cells (CD8^+^CD28^+^) (Figure 7B, C, E, G). Notably, activated CD8^+^ T cells were significantly increased on day 3 compared with baseline (12.34 ± 4.98% vs. 9.95 ± 4.51%, *p* < 0.05) (Figure 7G). At day 28, regulatory T cells (CD4^+^CD25^+^CD127^low^) were significantly increased relative to pre-ablation levels in the IRE group (2.40 ± 1.21% vs. 1.22 ± 0.80%, *p* < 0.05), and similarly in the RFA group (2.00 ± 1.44% vs. 1.30 ± 0.83%, *p* < 0.05) (Figure 7H).

## Discussion

In this prospective study, we longitudinally evaluated post-ablation tissue remodeling following IRE and RFA, with a primary focus on radiological changes of the ablation zone. Although ablation volume decreased over time in both groups, IRE was characterized by a faster and more heterogeneous remodeling process, whereas RFA demonstrated a slower, relatively shape-preserving contraction during follow-up. Overall treatment efficacy was comparable between IRE and RFA, with similar long-term local tumor control (LRFS at 6 months, 1 year, and 3 years: 90.9%, 81.8%, and 81.8% for IRE vs. 100%, 91.7%, and 78.6% for RFA). Although procedure-related complications were more frequent after IRE, they were generally manageable and no severe treatment-related adverse events occurred. Transient peripheral immune fluctuations were observed during follow-up, with a tendency toward more pronounced immune modulation after IRE.

Previous studies have described progressive volume reduction of the ablation zone following both IRE and thermal ablation. Dollinger et al. reported that the IRE ablation zone had decreased to 29% of its initial enhanced-CT image after about 4.7 months, consistent with findings reported by Kingham et al. 10, 11. In addition, a comparative imaging study by Scheck et al. demonstrated a more rapid reduction of the ablation zone following IRE compared with RFA during following-up 6. To minimize the influence of acute post-procedural changes such as hemorrhage and edema, post-ablation day 7 imaging was used as the reference point for longitudinal assessment 12. Venous-phase MRI was preferentially applied in patients without local recurrence, enabling more detailed delineation of ablation zone morphology compared with contrast-enhanced CT 13. Using this approach, a more pronounced early reduction in ablation zone volume was observed after IRE, with relative volumes decreasing to approximately 0.5 at 1 month and 0.11 at 3 months, compared with 0.76 and 0.49, respectively, after RFA. Beyond volumetric regression, distinct morphological remodeling patterns were also noted. IRE demonstrated a greater reduction in residual ablation-zone surface area during follow-up, particularly at 3 months, whereas RFA exhibited a more shape-preserving contraction pattern reflected by differences in the surface area-to-volume ratio. The distinct remodeling patterns may be related to fundamental differences in their mechanisms of tissue injury. Thermal ablation induces coagulative necrosis, resulting in protein denaturation and thermal fixation of the underlying tissue scaffold, which may lead to a more uniform, shape-preserving contraction of the ablation zone during resorption 5, 14, 15. In contrast, IRE primarily induces non-thermal cell death through apoptosis 3, accompanied by early and transient remodeling of the tumor microenvironment, including extracellular matrix softening and increased microvessel density and permeability 16. Preclinical studies have also shown that apoptotic cells induced by IRE are rapidly cleared through natural degradation and phagocytic processes during the early post-treatment period 17. Meanwhile, higher levels of liver regeneration associated factors, such as hepatocyte growth factor (HGF), have been reported in ablation zones following IRE compared with RFA, potentially contributing to enhanced tissue regeneration and distinct remodeling patterns 18. Collectively, these biological effects are thought to occur early after treatment and may influence subsequent tissue remodeling observed on longitudinal imaging, underscoring the importance of temporal dynamics in the healing process following ablation.

In terms of clinical outcomes, IRE and RFA achieved comparable ablation efficacy, as reflected by similar outcomes in LRFS, ORR, PFS, and OS during follow-up. However, differences were observed in safety profiles, with procedure-related bleeding events occurring more frequently in the IRE group. This difference may be related to the technical complexity of IRE, particularly the difficulty of achieving ideal parallel electrode alignment in complex lesions, which may lead to heterogeneous electric field distribution and focal vascular perturbation 19. Furthermore, the non-thermal nature of IRE preserves vascular patency, potentially predisposing to transient blood extravasation 18. In contrast, RFA is typically performed using a single electrode and induces coagulative necrosis, leading to thermal fixation of the ablation zone and intratumoral vessels, which may reduce the risk of bleeding during both electrode placement and the ablation process. Meanwhile, the needle track ablation process during the withdrawal of the ablation needle also reduces the risk of bleeding. Nevertheless, the bleeding events observed after IRE were generally manageable and remained within an acceptable safety range.

Accumulating evidence suggests that ablation-induced tissue damage actively engages the host immune system, which plays a critical role in coordinating tissue clearance, wound healing, and subsequent tumor surveillance. In the present study, dynamic immune fluctuations were observed in peripheral blood during the early post-ablation period (days 3-7), characterized by an increased proportion of activated T cells. Notably, IRE treatment tended to be associated with higher proportions of CD4^+^ T cells and activated CD8^+^ T cells, which may be related to the enhanced immunogenicity associated with non-thermal ablation mechanisms. By minimizing secondary thermal injury, IRE may better preserve tumor-associated antigens and damage-associated molecular patterns, in contrast to thermal ablation modalities such as RFA 14, where heat-induced protein denaturation may limit immunogenic signaling. At a later time point, an increase in circulating regulatory T cells (Tregs) was observed at approximately 1 month after ablation in both treatment groups. Regulatory T cells are widely recognized as key modulators of immune homeostasis, with well-established roles in tumor-associated immunosuppression 20, but also in limiting excessive inflammation and orchestrating tissue repair following injury 21. In this context, regulatory immune mechanisms may counterbalance ablation-induced immune activation, thereby constraining the durability of antitumor immune responses. The coexistence of transient immune activation and subsequent regulatory responses highlights the limited magnitude and durability of ablation-induced immune activation. The occurrence of local tumor progression and patient mortality in our cohort further underscores the complexity of post-ablation immune dynamics and suggests that immune activation elicited by local ablation alone may be insufficient to achieve sustained tumor control especially for patients with liver metastasis. This observation indicates that additional systemic approaches may be required to achieve durable disease control 16, 22. For the setting of curative-intent ablation, the delayed expansion of Tregs may also reflect an immune resolution phase associated with tissue repair and restoration of homeostasis.

Several limitations should be acknowledged. The relatively small and heterogeneous cohort, including both primary and secondary liver tumors, may limit the generalizability of the clinical, radiological, and peripheral immune findings. In addition, due to ethical and safety considerations, post-ablation tissue sampling was not performed, precluding direct assessment of local immune cell infiltration and tissue-level microenvironmental remodeling. Therefore, the observed peripheral immune alterations may not necessarily reflect intratumoral immune microenvironment changes and could instead represent systemic inflammatory or immunomodulatory responses following ablation.

Validation in larger, more homogeneous cohorts with integrated tissue-level analyses will be important to confirm these observations. Mechanistic investigations integrating imaging, histology, and immune profiling are needed to clarify the tissue repair processes underlying different ablation modalities and to establish direct correlations between longitudinal radiological evolution and histological remodeling. Future prospective studies are needed to explore whether distinct post-ablation radiological patterns are associated with long-term clinical outcomes.

## Conclusion

This study demonstrated distinct longitudinal radiological remodeling patterns of ablation zones following IRE and RFA, reflecting fundamental differences in their mechanisms of tissue injury. While RFA was associated with a more gradual, shape-preserving repair process and fewer procedure-related complications, IRE exhibited more pronounced and heterogeneous post-ablation remodeling with an acceptable safety profile. Despite these differences, both ablation modalities achieved comparable local tumor control and overall clinical outcomes. The observed immune fluctuations following IRE suggest potential biological differences between thermal and non-thermal ablation, warranting further investigation.

## Supplementary Material

Supplementary tables.

## Figures and Tables

**Table 1:** Baseline patient and tumor characteristics.

Characteristic	IRE (n = 11)^1^	RFA (n = 13)^1^	Overall (n = 24)^1^	P value^2^
Gender				> 0.9
Female	4 (36%)	4 (31%)	8 (33%)	
Male	7 (64%)	9 (69%)	16 (67%)	
Age	57.4 ± 6.8	57.5 ± 9.7	57.5 ± 8.3	> 0.9
BMI	22.4 ± 2.5	23.7 ± 3.7	23.1 ± 3.2	0.3
ECOG/Karnofsky performance status				0.6
ECOG 0	8 (73%)	11 (85%)	19 (79%)	
ECOG 1	3 (27%)	2 (15%)	5 (21%)	
Hepatitis status				0.8
HBV	3 (27%)	5 (38%)	8 (33%)	
HCV	1 (9.1%)	0 (0%)	1 (4.2%)	
None	7 (64%)	8 (62%)	15 (63%)	
Liver cirrhosis	5 (45%)	7 (54%)	12 (50%)	0.7
Tumor type				0.2
Hepatocellular carcinoma	5 (45%)	7 (54%)	12 (50%)	
Liver metastatic tumor (from colorectal cancer)	3 (27%)	4 (31%)	7 (29%)	
Liver metastatic tumor (from gastric cancer)	0 (0%)	2 (15%)	2 (8.3%)	
Liver metastatic tumor (from other cancer)	3 (27%)	0 (0%)	3 (13%)	
Number of lesions				0.8
1	8 (73%)	7 (54%)	15 (63%)	
2	2 (18%)	3 (23%)	5 (21%)	
3	0 (0%)	2 (15%)	2 (8.3%)	
4	1 (9.1%)	1 (7.7%)	2 (8.3%)	
Average maximum diameter (mm)	18.4 ± 6.6	19.4 ± 10.1	18.9 ± 8.5	0.8
Prior treatment before ablation				> 0.9
Chemotherapy	2 (18%)	3 (23%)	5 (21%)	
Embolization	1 (9.1%)	3 (23%)	4 (17%)	
Ablation	1 (9.1%)	1 (7.7%)	2 (8.3%)	
Surgery	4 (36%)	4 (31%)	8 (33%)	
None	3 (27%)	2 (15%)	5 (21%)	

^1^n (%); Mean ± SD. ^2^Fisher's exact test; Welch Two Sample t-test; Pearson's Chi-squared test

**Table 2:** Ablation-zone measurements and normalized residual fractions (vs day 7) by ablation modality.

Group	Outcome	N^1^	IRE (N = 12)^2^	RFA (N = 23)^2^	P value^3^
Absolute measurements (surface area)	Ablation-zone surface area at 7 days (mm²)	35	4,143.4 (3,351.1, 5,722.4)	5,360.3 (3,983.7, 6,270.1)	0.208
	Ablation-zone surface area at 1 month (mm²)	35	2,120.4 (1,690.3, 3,333.2)	4,145.5 (3,249.4, 5,188.4)	0.005
	Ablation-zone surface area at 3 months (mm²)	33	667.6 (462.1, 1,410.4)	3,202.6 (1,838.1, 3,740.7)	<0.001
Absolute measurements (volume)	Ablation-zone volume at 7 days (mm³)	35	15,301.5 (10,331.0, 20,161.0)	23,672.7 (14,064.7, 31,914.7)	0.031
	Ablation-zone volume at 1 month (mm³)	35	6,543.8 (4,846.6, 9,986.4)	16,337.0 (11,991.6, 25,511.0)	0.007
	Ablation-zone volume at 3 months (mm³)	33	1,075.4 (564.5, 3,251.0)	11,096.6 (6,247.8, 17,883.4)	<0.001
Normalized fractions (surface area)	Residual ablation-zone surface area fraction (1M/7D)	35	0.61 (0.40, 0.68)	0.82 (0.73, 0.88)	<0.001
	Residual ablation-zone surface area fraction (3M/7D)	33	0.20 (0.06, 0.35)	0.61 (0.50, 0.70)	<0.001
Normalized fractions (volume)	Residual ablation-zone volume fraction (1M/7D)	35	0.50 (0.35, 0.62)	0.76 (0.61, 0.85)	<0.001
	Residual ablation-zone volume fraction (3M/7D)	33	0.11 (0.03, 0.23)	0.49 (0.38, 0.65)	<0.001

^1^N: Number of non-progressive measurable lesions;^ 2^Median (Q1, Q3); ^3^Wilcoxon rank sum exact test

**Table 3:** Clinical outcomes and procedure-related adverse events.

Outcome	Overall (n=24)1	IRE (n=11)1	RFA (n=13)1	P value2
Local progression	4 (17%)	2 (18%)	2 (15%)	>0.9
Complications	5 (21%)	5 (45%)	0 (0%)	0.011
Hemorrhage	4 (17%)	4 (36%)	0 (0%)	0.031
Bile duct dilatation	1 (4%)	1 (9%)	0 (0%)	0.5

^1^n (%); ^2^Fisher's exact test. Note: Patients could experience more than one complication; therefore, complication subtypes are not mutually exclusive.

**Table 4:** Lesion risk classification and adjacent structures.

Characteristic	IRE (n=11)^1^	RFA (n=13)^1^	Overall (n=24)^1^	P value^2^
High-risk location	7 (64%)	6 (46%)	13 (54%)	0.4
Adjacent structure				0.062
Diaphragm	1 (9%)	1 (8%)	2 (8%)	
Gallbladder	0 (0%)	3 (23%)	3 (13%)	
GI tract	1 (9%)	1 (8%)	2 (8%)	
Hepatic hilum	2 (18%)	0 (0%)	2 (8%)	
Major vessels	3 (27%)	0 (0%)	3 (13%)	
None	4 (36%)	8 (62%)	12 (50%)	

^1^n (%); ^2^Pearson's Chi-squared test; Fisher's exact test.

## Abbreviations

IRE: irreversible electroporation
RFA: radiofrequency ablation
LRFS: local recurrence-free survival
MRI: magnetic resonance imaging
CT: computed tomography
ECG: electrocardiogram
LTP: local tumor progression
PFS: progression-free survival
OS: overall survival
ORR: objective response rate
mRECIST: modified Response Evaluation Criteria in Solid Tumors
SIR: Society of Interventional Radiology
IQR: interquartile range
NLR: neutrophil-to-lymphocyte ratio
HGF: hepatocyte growth factor
Tregs: regulatory T cell
