# Supplementary material for: Longitudinal Radiological Remodeling after Irreversible Electroporation Versus Radiofrequency Ablation: A Prospective Comparative Study (The LIRA Study)
Source: Int J Med Sci. 2026 Mar 17;23(4):1470–81. doi: 10.7150/ijms.133300 (PMC13048883; doi:10.7150/ijms.133300)
Supplement: Supplementary file 1 — Supplementary tables. [file ijmsv23p1470s1.pdf]

Supplementary Table 1. Local Tumor Progression in Primary and Metastatic Liver Tumors by Ablation Method

	Primary			Metastatic		
**Status**	**Overall** N = 12[1]	**IRE** N = 5[1]	**RFA** N = 7[1]	**Overall** N = 12[1]	**IRE** N = 6[1]	**RFA** N = 6[1]
**Local Tumor Progression Status**						
No progression	11 / 12 (91.7%)	5 / 5 (100.0%)	6 / 7 (85.7%)	9 / 12 (75.0%)	4 / 6 (66.7%)	5 / 6 (83.3%)
Progression	1 / 12 (8.3%)	0 / 5 (0.0%)	1 / 7 (14.3%)	3 / 12 (25.0%)	2 / 6 (33.3%)	1 / 6 (16.7%)

[1]n / N (%)

Supplementary Table 2. Longitudinal changes in peripheral immune-related blood parameters after ablation.

Group	Parameter	Pre-ablation[1]	Day 1[1]	Day 3[1]	Day 7[1]	Week 2[1]	Week 4[1]
IRE	**WBC ($\times 10^9$/L)**	5.05 (3.33, 8.69)	7.57 (5.95, 10.65)	6.05 (5.15, 9.85)	5.30 (3.41, 7.00)	5.56 (3.10, 5.72)	4.55 (2.90, 5.51)
	**Neutrophils ($\times 10^9$/L)**	3.03 (1.90, 6.00)	6.37 (4.53, 8.80)	4.42 (3.42, 7.09)	3.53 (2.28, 4.80)	3.31 (1.92, 3.73)	2.28 (1.70, 3.15)
	**Lymphocytes ($\times 10^9$/L)**	1.38 (0.84, 1.54)	0.82 (0.47, 1.19)	0.90 (0.74, 1.34)	1.00 (0.71, 1.38)	1.26 (0.87, 1.50)	0.85 (0.80, 1.48)
	**NLR**	2.41 (2.11, 3.75)	8.47 (5.21, 11.09)	4.15 (3.35, 7.35)	3.90 (1.91, 4.96)	2.35 (2.21, 3.35)	1.88 (1.35, 2.56)
RFA	**WBC ($\times 10^9$/L)**	4.33 (3.78, 4.80)	7.33 (4.64, 7.65)	5.19 (4.54, 8.37)	4.24 (3.12, 4.31)	4.40 (3.60, 5.98)	3.50 (3.00, 4.77)
	**Neutrophils ($\times 10^9$/L)**	2.81 (2.28, 3.23)	6.20 (4.05, 6.70)	3.48 (3.38, 6.26)	2.59 (2.11, 3.00)	3.02 (2.38, 4.38)	2.27 (1.73, 2.69)
	**Lymphocytes ($\times 10^9$/L)**	1.00 (0.75, 1.51)	0.71 (0.35, 0.91)	0.87 (0.67, 1.04)	0.80 (0.70, 0.95)	0.97 (0.78, 1.10)	1.00 (0.77, 1.07)
	**NLR**	2.81 (1.99, 3.90)	11.18 (5.54, 16.65)	5.43 (4.16, 8.18)	3.59 (2.95, 4.35)	3.51 (3.05, 4.58)	2.20 (1.95, 3.80)

[1]Median (Q1, Q3)
